# Genetic structure along an altitudinal gradient in *Lippia origanoides*, a promising aromatic plant species restricted to semiarid areas in northern South America

**DOI:** 10.1002/ece3.360

**Published:** 2012-09-27

**Authors:** Nelson Enrique Vega-Vela, María Isabel Chacón Sánchez

**Affiliations:** Facultad de Agronomía, Universidad Nacional de ColombiaCarrera 30 # 45 – 03 Edificio 500, Bogotá D.C., Colombia

**Keywords:** AFLP, elevation gradient, genetic differentiation, genetic similarity, isolation by distance

## Abstract

The genetic diversity and population structure of *Lippia origanoides*, a species of the Verbenaceae family that shows promise as a crop plant, was investigated along an altitudinal gradient in the basin of the Chicamocha River in northeastern Colombia. The economic importance of the species, quality of its essential oils, and the fact that it is restricted to some few semiarid areas in northern South America may put the species at risk in a scenario of uncontrolled harvest of natural populations. *Lippia origanoides* was sampled along an altitudinal gradient from 365 to 2595 m.a.s.l. throughout Chicamocha River Canyon, a semiarid area in northeastern Colombia. Genetic diversity was assessed by means of AFLP markers. The number of AFLP loci (355) and the number of individuals sampled (173) were sufficient to reliably identify four populations at contrasting altitudes (*F*_*ST*_ = 0.18, *P-value* < 0.0000), two populations in the lower basin, one population in the medium basin, and one population in the upper basin, with a low level of admixture between them. In average, genetic diversity within populations was relatively high (*Ht* = 0.32; *I* = 0.48); nevertheless, diversity was significantly reduced at higher altitude, a pattern that may be consistent with a scenario of range expansion toward higher elevations in an environment with more extreme conditions. The differences in altitude among the basins in the Chicamocha River seem to be relevant in determining the genetic structure of this species.

## Introduction

Plant species that show tolerance or resistance to extreme conditions, such as those associated with arid and semiarid areas and that, additionally, are important for agricultural and/or industrial applications, are of interest for population genetics studies, not only because these species may be pre-adapted to the consequences of climate change (e.g., more prolonged dry seasons) but also because their uncontrolled exploitation from the wild may impose a risk for their survival.

An example of these kinds of species is *Lippia origanoides* H.B.K., an aromatic plant species of the Verbenaceae family that has promise for the quality and potential industrial use of its essential oils. The species occurs in northern South America (de Campos et al. 2011; Pascual et al. 2001; Suárez et al. 2008), in restricted and isolated areas with xerophytic and subxerophytic conditions in Colombia and Venezuela (Albesiano 2003; Albesiano and Rangel-Ch 2006; Parra and Rodríguez 2007). In addition to its use, *L. origanoides* is a species of scientific interest because it shows high tolerance and resistance to the stress imposed by environmental factors typical of arid and semiarid environments (Parra and Rodríguez 2007; Antolinez-Delgado and Rodríguez 2008; Camargo and Rodríguez 2008). In Colombia, *L. origanoides* has been reported in semiarid areas of the northeast (in the departments of Santander and Boyacá, specially, in the canyon of the Chicamocha River), the south (departments of Cauca and Nariño), and the Atlantic coast (department of Magdalena) (Albesiano 2003; Albesiano and Rangel-Ch 2006; Suárez et al. 2008; Stashenko et al. 2010).

*Lippia origanoides* has an important phytochemical variation represented in three chemotypes according to the major compounds present in their essential oils (chemotype A: *p-cymene*, chemotype B: *carvacrol*, and chemotype C: *thymol*) (Stashenko et al. 2010). The bioactivity of the extracts and essential oils of these chemotypes has been evaluated with promising results in assays against *Spongospora subterranea* (Bittara et al. 2009), *Sclerotium rolfsii*, *Macrophomina phaseolina* (Ulacio et al. 2008), *Rhizoctonia solani*, *Bipolaris maydis* (Rodríguez and Sanabria 2005), *Mycosphaerella fijiensis* (Vargas et al. 2009), *Mycobacterium tuberculosis* (Bueno-Sánchez et al. 2009), *Candida* sp. (dos Santos et al. 2004; Henao et al. 2009; Oliveira et al. 2007), *Leishmania chagasi*, *Trypanosoma cruzi* (Celis et al. 2007; Escobar et al. 2010), *Escherichia coli*, *Staphylococcus aureus*, *Salmonella* sp., and others microorganisms (dos Santos et al. 2004; Henao et al. 2009; Oliveira et al. 2007; Ramírez et al. 2009). Additionally, these essential oils have shown antioxidant activity (Celis et al. 2007; Muñoz-Acevedo et al. 2009), repellent activity against *Tribolium castaneum* and *Sitophilus zeamais* (Nerio et al. 2009; Olivero-Verbel et al. 2009), antiviral effect on Dengue Virus and Yellow Fever Virus (Meneses et al. 2009a,b), and DNA protective effect against bleomycin-induced genotoxicity (Vicuña et al. 2010). These results show that the extracts and essential oils of *L. origanoides* are of broad spectrum (Celis et al. 2007; Stashenko et al. 2010), with potential uses in various industries such as food, cosmetics, pharmaceutical and agricultural, and are suitable as a source of phytomedicines (Bueno-Sánchez et al. 2009; Stashenko et al. 2010; Vicuña et al. 2010). Nowadays, *L. origanoides* is only found in wild habitats and no cultivated varieties have been developed, which poses it a risk in a scenario of uncontrolled harvesting for industrial use.

Very little is known about basic genetic aspects of this species. In a recent study, de Campos et al. (2011) reported the haploid number of *L. origanoides* as *n* = 12. Nevertheless, additional studies are needed to confirm its monoploid number and ploidy (de Campos et al. 2011). On the other hand, the only study that describes aspects of population genetic diversity in this species is the one of Suárez et al. (2008). The authors studied the spatial genetic structure in a population of *L. origanoides* from the lower basin of the Chicamocha River in Colombia. This population exhibited relatively high levels of genetic diversity (Suárez et al. 2008) similar to those reported in other species of the genus *Lippia* with similar geographic distributions and life histories (Viccini et al. 2004).

The canyon of the Chicamocha River is a semiarid zone that encompasses an area of about 300,000 hectares that expands between the departments of Santander and Boyyacá in northeastern Colombia, with elevations ranging from 300 to 2600 meters above the sea level (m.a.s.l.), in a topography of steep slopes (>60°) and shallow soils (Albesiano 2003; Albesiano and Rangel-Ch 2006). At low altitudes, *L. origanoides* is relatively abundant, because of its ability to tolerate stress and its phenotypic plasticity (Parra and Rodríguez 2007; Antolinez-Delgado and Rodríguez 2008; Camargo and Rodríguez 2008), and other factors such as low grazing pressure (Albesiano and Rangel-Ch 2006; Suárez et al. 2008). In contrast, we observed that the abundance of *L. origanoides* becomes reduced at higher altitude, presumably, as a result of range expansion and decreased ability of the species to survive under high altitude conditions, which could lead to decreased genetic variability.

The objective of this study was to evaluate the genetic structure of *L. origanoides* along an elevation gradient (365–2595 m.a.s.l.) at the lower, medium, and upper basins of the Chicamocha River, a semiarid area in Colombia, using AFLP molecular markers. Our ultimate aim is to investigate the processes that influenced the current distribution of *L. origanoides* in the canyon, mainly in those populations at higher elevations. For this purpose, we analyzed a sample of 173 individuals from the lower, medium, and upper basins of the canyon, using 355 AFLP loci, and applied a variety of approaches for assessing genetic structure and diversity with dominant markers.

## Materials and Methods

### Study species

**Figure 1:**
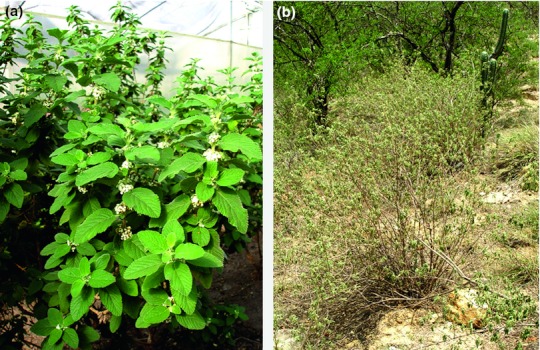
Individual of *Lippia origanoides* growing under: (a) greenhouse and (b) natural conditions.

*Lippia origanoides* is an aromatic shrub, erect and branched, which grows to 3 m in height and belongs to the Verbenaceae family (Fig. 1). This shrub has simple and opposite leaves of variable sizes due to possible physiological and morphological adaptations in response to exposure to light (Parra and Rodríguez 2007), with inflorescences characterized by white flowers, small and pedicellate (4 mm in size) (de Campos et al. 2011), and a high yield of dried fruits and seeds per plant. *Lippia origanoides* has a pungent odor similar to the spice known as oregano, due to the presence of secondary metabolites, such as *carvacrol*, *thymol*, *p-cymene*, among other phenolic compounds responsible for the particular aroma and flavor of this spice (Calpouzos 1954; Arcila-Lozano et al. 2004). Because of this, *L. origanoides* is known as “oregano de monte” and it is classified within the group of species known as “oregano”, with extracts and essential oils of comparable chemical compositions, exhibiting similar antioxidant, antimicrobial, antigenotoxic, and antiparasitic activities (Calpouzos 1954; Pascual et al. 2001; Arcila-Lozano et al. 2004).

### Sample collection

**Figure 2:**
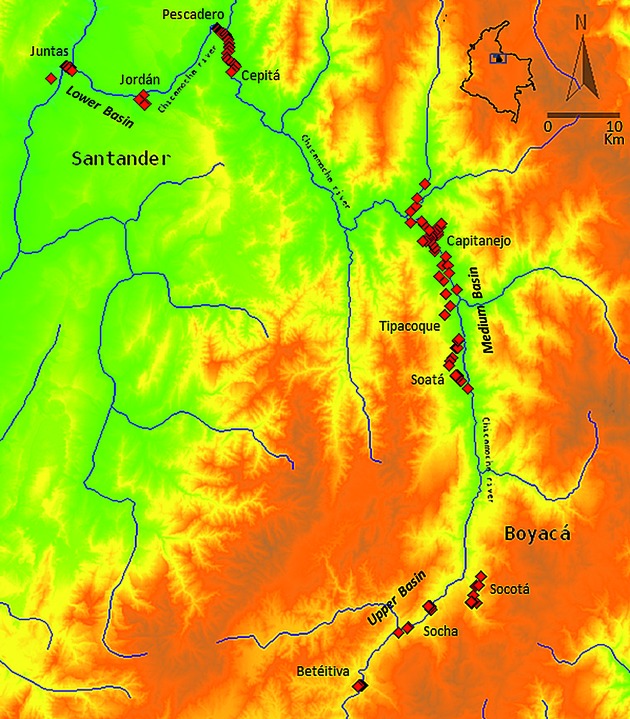
Map of the study area in the canyon of the Chicamocha River, indicating sampling sites. The map shows the major localities of the collection in the departments of Santander and Boyacá, Colombia. The red dots indicate the geo-referenced collection points from each individual. Samples from 73, 66, and 34 individuals were collected in the lower, medium, and upper basin of the canyon, respectively.

Fresh plant material for 173 individuals was collected in the canyon of the Chicamocha River between August and November, 2009. Sampling was carried out according to Suárez et al. (2008). The sampling area comprised the lower, medium, and upper basins of the Chicamocha River (see Fig. 2), in elevations that ranged from 365 to 2595 m.a.s.l. For each sample, 20–30 young leaves were collected in zip-lock plastic bags containing silica gel and stored at −80°C until DNA extraction. For each sample collected, latitude, longitude, and elevation data were recorded using a GPS from GARMIN (USA). Vouchers from each locality were deposited at the Herbario Nacional Colombiano (COL) (accession No. 550395 – 550408).

### DNA extraction

High-quality DNA suitable for genetic analyses in *L. origanoides* was obtained using the chemical denaturing properties of the guanidinium cation and the silica, in a protocol optimized to aromatic and medicinal plants (Vega-Vela and Chacón 2011). Basically, 150 mg of dried tissue for each sample was ground in liquid nitrogen and immediately transferred into a sterile 2-mL Eppendorf tube containing 800 mL of buffer [2% CTAB w/v, 1.5 mol/L NaCl, 20 mM EDTA, 100 mmol/L Tris-HCl pH 8.0, and 1% β-mercaptoethanol]. The samples were homogenized by gently inversion and incubated for 30 min at 65°C. Subsequently for each sample, 800 μL of chloroform:isoamyl alcohol (24:1) was added, mixed by vigorous shaking to achieve an emulsion, and centrifuged at 14,400 × g for 10 min. The supernatant was transferred into a sterile 2-mL Eppendorf tube and 200 μL of isopropanol at −20°C was added and mixed gently by inversion. Afterward, 1 mL of Gdm -salt solution [Guanidinium Hydrochloride (GdmHCl) Solution: 4 mol/L GdmHCl dissolved in sterile distilled water] was added and quickly mixed by inversion for 5 min. The sample was transferred to a silica-based column (EconoSpin™ All-in-1; Epoch Biolabs, Missouri City, Texas) and centrifuged at 10,000 × g for 5 min; the flow-through was discarded. The membrane was washed twice by adding 0.5 mL of 90% ethanol and centrifuged at 10,000 × g for 5 min. In a new sterile collection tube, 100 μL of low salt TE buffer pH 8.0 preheated at 65°C was added directly in the center of the membrane and incubated for 5 min. The flow-through with the DNA was obtained by centrifugation at 10,000 × g for 1 min. Finally, 2 μL of RNase A (1 μg/μL) was added to each sample and incubated at 37°C for 15 min. The quality of each sample was checked in a 1% agarose gel. Additionally, all samples were quantitated using a NanoDrop™ 2000 spectrophotometer (Thermo Fisher Scientific Inc., Waltham, Massachusetts). The absorbance measurements to qualify the purity of the extracted DNA were A_260_/A_280_ = 1.80 and A_260_/A_230_ = 1.87 (Vega-Vela and Chacón 2011).

### AFLP fingerprinting

The kit AFLP® Analysis System I (Invitrogen™, Carlsbad, California) was used to amplify five selective primer combinations (*EcoRI* – AGG/*MseI* – CTC, *EcoRI* – ACA/*MseI* – CAT, *EcoRI* – AGG/*MseI* – CAT, *EcoRI* – ACT/*MseI* – CAT, and *EcoRI* – AAC/*MseI* – CTA) that according to preliminary results are informative in *L. origanoides* (N. E. Vega-Vela, unpubl. data). The AFLP amplifications were carried out as recommended by the manufacturer. The visualization of the products was performed following the manufacturer's instructions, although the staining of 6% PAGE was performed in silver nitrate. The gels were documented with a scanner Epson Perfection® 4490 Photo (Epson America, Long Beach, California); each image was optimized and scored using GIMP 2.6 and EasyPen i405 (Genius, KYE Systems America Corporation, Miami, Florida). This procedure of *digital-scoring* was performed by painting a black line on the bands indicating their presence and a white line indicating the absence of the fragment, which improved automated processing for obtaining the presence/absence or 1/0 matrix with Cross Checker 2.91 (Buntjer and Otsen 1999) from polyacrylamide gels.

### Analysis of data

We scored fragments in the range between 150 and 1000 bp. Two levels of polymorphism were assessed for all the analyses conducted, 0.99 and 0.95, and no significant differences were observed. The genetic relationships among individuals were evaluated by a PCA (Principal Coordinates Analysis) using the distance matrix under the method proposed by Huff et al. (1993), based on the algorithm of Orloci (1978) and implemented in GENALEX 6.4 (Peakall and Smouse 2006). A 3D PCA graphic was performed using the *plot3d* command of the RGL library (Adler and Murdoch 2011) in the program R (R Development Core Team 2011). Additionally, we used DARWIN 5 (Perrier and Jacquemoud-Collet 2006) to calculate matrices of pairwise Dice and Jaccard dissimilarity indices. Dendrograms were constructed using hierarchical clustering by the UPGMA method (Unweighted Pair Group Method with Arithmetic Mean). Other indices and genetic distances were checked, but no significant differences were observed.

In most genetic studies, the number of markers and the number of individuals sampled are defined either arbitrarily or by available resources. However, in genetic structure studies, this may have a great effect on the validity and reliability of the inferred clusters. For this reason, we used the method reported by Medina et al. (2006) for determining whether the number of individuals and markers used were enough to describe the genetic structure in our sample. Basically, the SESIM code calculates for different combinations of number of individuals by number of markers, the standard error of the mean similarity index (SESIM), or distance measure by randomly subsampling matrices (Medina et al. 2006). The Jaccard similarity index and 1000 simulations were used; SESIM-values close to zero are preferred because it suggests consistency in the clustering.

We estimated the appropriate number of groups or partitions (populations or subpopulations), in addition to the level of admixture between the groups, and assigned each individual to each of the inferred populations using two Bayesian approaches implemented in the programs STRUCTURE 2.2 (Pritchard et al. 2000; Falush et al. 2007) and BAPS 5.3 (Corander and Marttinen 2006; Corander et al. 2008a,b).

STRUCTURE is widely used for inferring population structure, assigning individuals to populations, identifying migrants, and admixed individuals. STRUCTURE implements a model-based clustering based on a Bayesian Markov Chain Monte Carlo (MCMC) approach, in which there are K populations and the individuals are assigned to these populations probabilistically. We used a model of ancestry with no admixture and a model of allele frequencies uncorrelated or independent, suitable for dominant molecular markers (Ehrich et al. 2007). Ten runs were conducted for each value of K ranging from 1 to 10. The length of burnin period was 1 million with 1 million MCMC replicates after burnin. All runs were carried out at the BIOPORTAL of the University of Oslo (http://www.bioportal.uio.no). Similarity among runs was estimated according to Rosenberg et al. (2002), and *ΔK* as the mean of the absolute values of the second order rate of change of *L(K)* (Ln *P(D)* in STRUCTURE output) divided by the standard deviation according to the methodology proposed by Evanno et al. (2005). Similarity and *ΔK* were calculated using an R-script “*Structure-sum-2011*” available from http://uit.no/ansatte/organisasjon/ansatte/person?p_document_id=41186&p_dimension_id=88165 (Rosenberg et al. 2002; Evanno et al. 2005; Ehrich 2006; Ehrich et al. 2007).

Similarly, we used BAPS (Bayesian Analysis of Population Structure) to infer the genetic structure in our data considering the geographic information collected in the field. Briefly, BAPS treats both K and allele frequencies as random variables, and returns one optimal partitioning and the probability of the optimal K. In order to strengthen the inferences, we used as prior the geographic coordinates of the sampled individuals. Spatial clustering and admixture of individuals based on mixture clustering were calculated using a vector of values of K from 2 to 20 and 20 replicates. Voronoi tessellation and admixture clustering graphics were obtained to indicate the optimal partitioning or potential population structure and the level of admixture within the clusters inferred from the canyon of the Chicamocha River.

Genetic diversity within the clusters inferred with the methodologies described above was estimated using different methods widely applied to dominant markers. The average number of pairwise differences between individuals within clusters (Kosman 2003) was calculated using the R-script AFLPDAT (Ehrich 2006). Using the software AFLPSURV (Vekemans et al. 2002), the allelic frequencies were computed according to two methods: (1) a square root method (Nei 1987) and (2) a Bayesian method with non-uniform prior distribution of allele frequencies (Zhivotovsky 1999), and the expected heterozygosity or Nei's gene diversity (Nei 1973) under HWE was estimated according to Lynch and Milligan (1994). A Bayesian approach implemented in HICKORY (Holsinger et al. 2002) under the *full* model was also used for estimating genetic diversity, defined as the average panmictic heterozygosity within each population or cluster, without assuming HWE (Zhivotovsky 1999). This software uses MCMC and implements a combination of slice sampling and a single-component Metropolis-Hastings sampler, with a Dirichlet distribution. *DIC* and *Dbar* had the smallest values in the *full* model, which was taken as criteria to choose this model, according to the HICKORY manual (Holsinger et al. 2002). We used the *full* model with the sampler parameters configured as burn-in = 20,000, sample = 200,000, thin = 20. Additionally, the percentage of polymorphic loci (*P*) and Shannon's information index (*I*) (Shannon and Weaver 1949) was calculated using POPGENE 1.32 (Yeh and Boyle 1997) and they were used as another measure to describe the genetic diversity.

Two approaches for determining the level of genetic differentiation among populations or clusters were used: (1) θ^II^, a measure of the amount of genetic differentiation among contemporaneous populations and estimated by HICKORY, and (2) AMOVA, analysis of molecular variance among populations using pairwise difference as distance method and 100,000 permutations for support, implemented in ARLEQUIN 3.5 (Excoffier and Lischer 2010).

## Results and Discussion

### Sample collection

*Lippia origanoides* is an aromatic and medicinal species promising for the quality of its essential oils and the broad activity spectrum that these have shown against pathogenic microorganisms, relevant to agriculture and human health (Pascual et al. 2001; Arcila-Lozano et al. 2004; Stashenko et al. 2008, 2010). In Colombia, it has been reported in semiarid areas where specimens have been collected for the study of their phytochemical variation (Stashenko et al. 2008, 2010; Vicuña et al. 2010).

In this study, 173 samples were collected throughout the lower, medium, and upper basins of the Chicamocha River (see Fig. 2), where the species exhibits particularly high phytochemical variation (Stashenko et al. 2010). In the lower basin (elevation: 300–1000 m.a.s.l.), 73 individuals were collected at three locations (Juntas, Jordán, and Pescadero – Cepitá, Santander, COL) in which previous papers suggested its existence (Albesiano 2003; Albesiano and Rangel-Ch 2006; Suárez et al. 2008). The greatest distance among any pair of individuals collected in this basin was 27 km. In the medium basin (elevation: 1000–2000 m.a.s.l.), 66 individuals were collected from the municipalities of Capitanejo (Santander, COL) and Soatá (Boyacá, COL) according to previous reports (Albesiano 2003; Albesiano and Rangel-Ch 2006). The greatest distance among any pair of individuals collected in this basin was 33 km. The topography within this basin suggests that there are no strong geographic barriers to gene flow because here the canyon reaches its greatest extent (see Fig. 2), forming a valley or dale through which it is likely that air streams circulate over long distances, as well as birds, insects, etc., which are likely to be important in pollen and seed dispersal. This situation was different to what was observed in the upper and lower basins, where the mountains and abrupt changes in the river flow probably constitute important barriers to gene flow and influence the shaping of the genetic structure of these populations (see Fig. 2, areas Jordan – Cepitá, Cepitá – Capitanejo, and Soatá – Socotá). In the upper basin (elevations >2000 m.a.s.l.), a total of 34 individuals were collected and the greatest distance among any pair of individuals collected was 16 km. In this basin, the abundance of *L. origanoides* was significantly lower than in the lower and medium basins, probably because in this area, suboptimal conditions for the development of the species are found, which may be determinant in the distribution of the species. Small patches with very few individuals per patch were typically found in this area of the canyon, which was reflected in the smaller number of individuals collected.

The greatest distance among any pair of individuals in the whole sample was approximately 105 km between the sites known as Juntas in Villanueva in the lower basin (Santander, COL) and Betéitiva in the upper basin (Boyacá, COL). The elevation range covered during the sampling was between 365 and 2595 m.a.s.l. for the entire canyon. On average, elevations for each basin were: (1) 532 m.a.s.l. for the lower basin, (2) 1440 m.a.s.l. for the medium basin, and (3) 2350 m.a.s.l. for the upper basin.

### Genetic relationships among individuals

Using five selective primer combinations, 355 AFLP loci were amplified and unambiguously scored using a manual scoring procedure for each allele in each locus (presence/absence of fragment) on a digital image (see Materials and Methods); 244 (68.7%) and 227 (63.9%) markers were polymorphic at 0.99 and 0.95, respectively. According to Lynch and Milligan (1994), in our study, the level of polymorphism suitable for estimation of diversity and genetic differentiation would be 0.983 (using the 3/*N* criterion as 1−(3/*N*), where *N* is the number of samples) (Lynch and Milligan 1994). However, all the analyses were performed using two sets of data configured at the level of polymorphism of 0.99 and 0.95 and no significant differences were observed, probably due to the high number of polymorphic markers (Lynch and Milligan 1994; Krauss 2000; Kosman and Leonard 2005).

**Figure 3:**
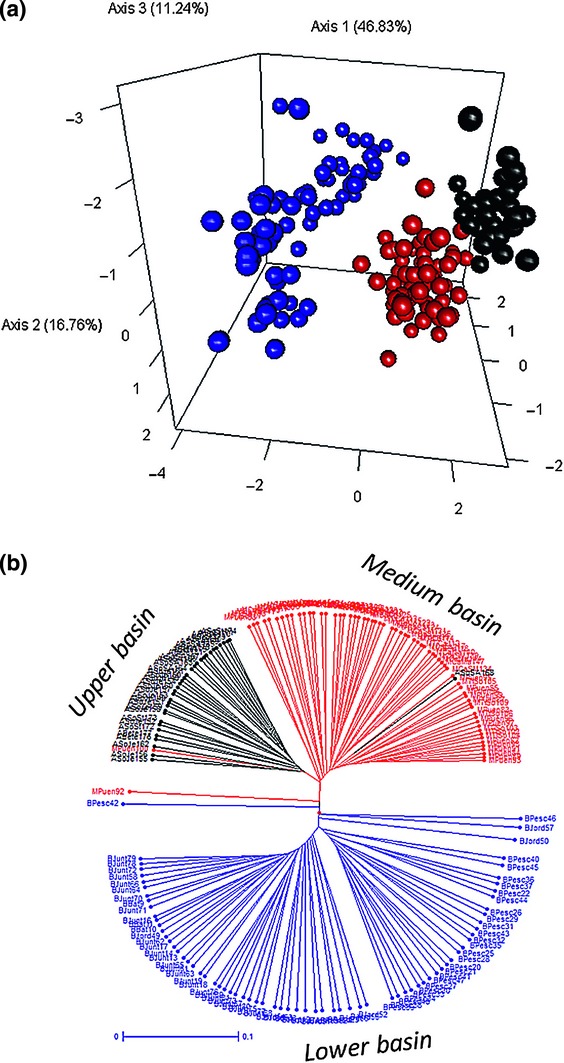
3D PCA graphic and dendrogram showing the genetic relationships of the individuals collected in the canyon of the Chicamocha River. (a) The first three axes of PCA explained a 74.8% of the total variation in the data. Individuals collected in the lower basin are shown in blue, individuals from the middle basin in red, and individuals from the upper basin of the canyon of the Chicamocha River in black. The 3D PCA used the method of Distance-Standardized implemented in GENALEX. (b) The dendrogram built using Dice's coefficient and Unweighted Pair Group Method with Arithmetic Mean revealed an apparent clustering of individuals from the same basin. Similar to what was observed in the 3D PCA, the dendrogram indicated a higher genetic heterogeneity among individuals from the lower basin with respect to individuals from the medium and upper basins. In the diagrams, there is low mixture of individuals between groups, indicating a discrete distribution of populations and a clear distinction between individuals from different basins.

**Figure 4:**
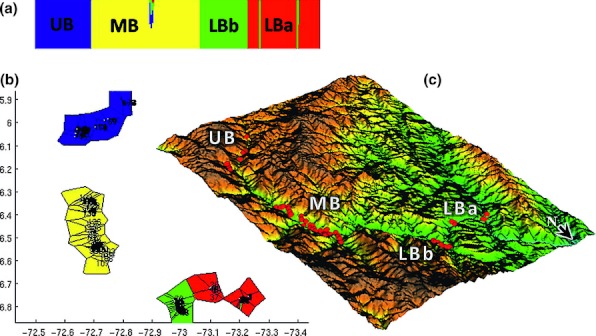
Admixture analysis and spatial clustering analysis using a Voronoi tessellation in BAPS. Spatial clustering analysis returned an optimal partition value of *K* = 4, which allowed determining four populations: (i) UB: population from the Upper Basin, (ii) MB: population from the Medium Basin, (iii) LBa: population from the Lower Basin A (Juntas and Jordán), and (iv) LBb: population from the Lower Basin B (Pescadero and Cepitá). In (a) Admixture analysis indicated low levels of admixture among populations. (b) According to Corander et al. (2008b), the spatial information increases the power to detect population structure. The Voronoi tessellation graphic shows a biologically relevant scenario in which populations are distributed discretely throughout the canyon. (c) A 3D model of the Chicamocha canyon built using GRASS (GRASS Development Team 2010) and QGIS (Quantum GIS Development Team 2011) on the SRTM 90-m Digital Elevation Data (Jarvis et al. 2008). The topography of the area probably determines the distribution of the species. The model points out the populations. Individuals are indicated as red dots.

A pairwise individual-by-individual genetic distance matrix was built following the method of Huff et al. (1993) and was used to perform a 3D PCA (Principal Coordinate Analysis using 3D graphic). It can be seen in Figure 3a that individuals tend to group into clusters according to their site of collection. Individuals from the upper basin (UB) formed a compact group and apparently are more closely related to individuals from the medium basin (MB). This suggests that individuals collected at elevations higher than 1000 m.a.s.l. tend to share more alleles, which may be in part due to similar ecological conditions for adaptation in these kinds of environments (Byars et al. 2009). Conversely, the individuals from the lower basin (LB) formed an independent and more dispersed cluster, which agrees with greater genetic heterogeneity within this cluster. According to Suárez et al. (2008), the individuals from the lower basin of the Chicamocha River, between Pescadero and Cepitá (Santander, COL) (subpopulation LBb in our study, see Figure 4), behave as a continuously distributed population. The remaining individuals that were collected in other localities from the lower basin (Juntas and Jordán, Santander, COL) probably constitute another subpopulations (LBa), which would explain the pattern observed in the 3D PCA (Fig. 3a).

Different indices of dissimilarity and genetic distances were calculated with similar results to those obtained in the 3D PCA. In Figure 3b, the dendrogram calculated with the Dice's coefficient and clustered by UPGMA revealed a pattern of clustering by collection site and altitude. In this diagram, and similar to the 3D PCA, the samples are grouped according to the basin where they were collected. It can also be observed that there seems to be low level of mixture between groups or basins, probably as a result of a discrete distribution determined by geographic barriers between populations in the canyon of the Chicamocha River. In addition, demographic processes similar to isolation by distance determined by the breeding system and seed dispersal in *L. origanoides* (Suárez et al. 2008) may be acting.

### Inference of the genetic structure – bayesian method: choosing K

According to Evanno et al. (2005), the inference of populations in a sample strongly influences further estimations of genetic diversity and differentiation. Graphic methods, such as 2D and 3D PCA, dendrograms, etc., have been widely used for determination of populations or groups based on multiple indices of dissimilarity and information about geographic origin, which has been adopted as a biologically significant approach (Krauss 2000; Evanno et al. 2005; Kosman and Leonard 2005). However, these methods are not fully recommended because of poor statistical support (Evanno et al. 2005; Bonin et al. 2007). For this reason, in this study, the inference of the genetic structure and subsequent estimation of genetic diversity and differentiation were also done with alternative approaches to detect statistically strong or subtle signs of population genetic structure (Bonin et al. 2007).

Using the software BAPS, we found that the optimal number of partitions was *K* = 4 (probability = 1), with low level of admixture between clusters or populations (see Fig. 4a), suggesting that each individual belongs undoubtedly to one group or partition in which it has its ancestral source. Spatial clustering, summarized in the Voronoi tessellation graphic (see Fig. 4b), indicated that populations are discretely distributed throughout the space in the canyon of the Chicamocha River, and there is correspondence between geographic location of individuals and their cluster membership. Therefore, the geographic conditions of each of the basins (e.g., contrasting elevations; see Fig. 4c) seem to be relevant in determining the genetic structure of this species in the canyon, although probably other processes that depend on the reproductive system and seed dispersal of the species are also acting. However, little is known about how the species reproduces and disperses; this is a subject that deserves further research in order to understand the genetic landscape of *L. origanoides* in the canyon of the Chicamocha River (Suárez et al. 2008).

**Figure 5:**
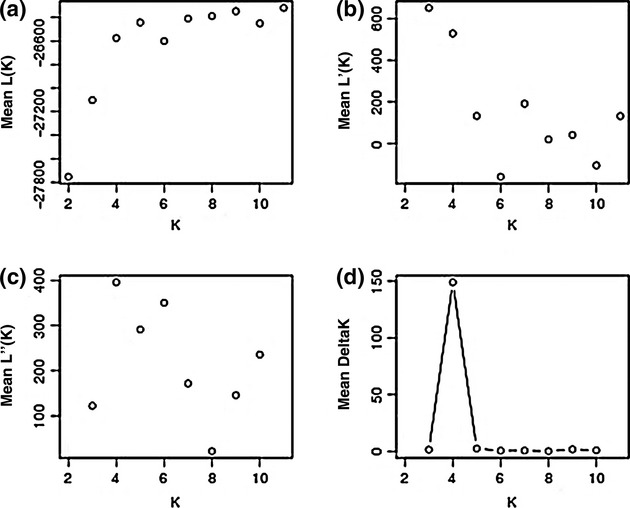
Population structure analysis using the methodology described by Evanno et al. (2005). The figure shows the four steps for the graphic method described by Evanno et al. (2005), allowing the detection of the optimal *K*. (a) Mean *L(K)*, over 10 runs for each *K*, (b) Mean *L′(K)*, rate of change of the likelihood distribution, (c) Mean *L″(K)*, absolute values of the second order rate of change of the likelihood distribution, and (d) *ΔK*. *K* = 4, was the optimal K for our data. Note that *L(K)* increased after achieving the optimal value of K, and the height of K = 4 indicates a strong signal of genetic structure detected by STRUCTURE.

**Figure 6:**
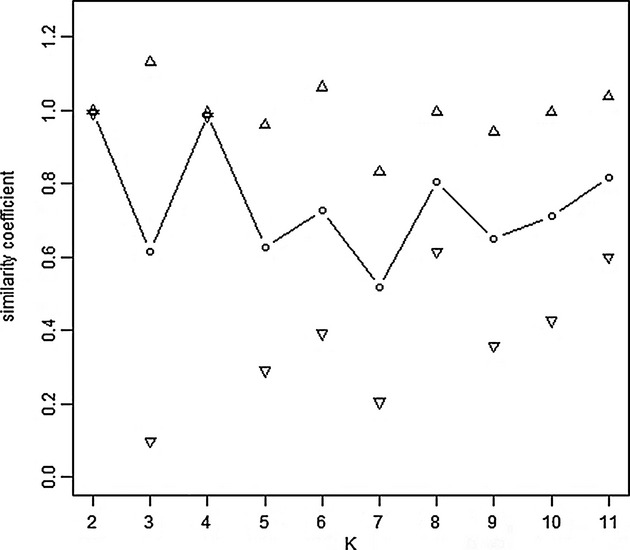
Similarity coefficient using the methodology proposed by Rosenberg et al. (2002). The figure shows the average coefficient of similarity for each *K*, with standard deviation. Note that the standard deviation in *K* = 2 and *K* = 4 were smaller, indicating that the genetic structure detected by STRUCTURE was similar throughout replicates (10 runs).

**Figure 7:**
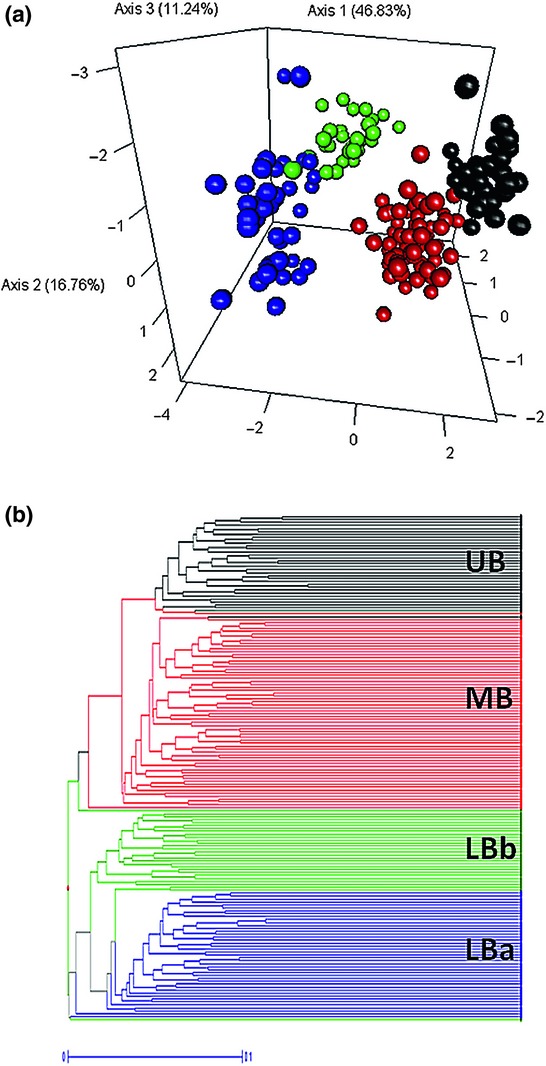
3D PCA and dendrogram showing the genetic relationships among individuals collected in the canyon of the Chicamocha River, using *K* = 4. (a) 3D PCA displaying the four populations identified in the Bayesian analysis. Compared to Figure 3, we found that despite having poor statistical support, the statistical graphic methods suggests the same groups or populations present in the canyon. (b) Dendrogram calculated with Jaccard's coefficient and Unweighted Pair Group Method with Arithmetic Mean indicating the genetic relationships among individuals and populations in the Chicamocha Canyon.

Similar results were obtained with STRUCTURE. It can be seen in Figure 5 that the modal value of the distribution of *ΔK* was located at *K* = 4, which in our data suggests that there are four groups or populations, confirming the results obtained previously with BAPS. Furthermore, in accordance with Evanno et al. (2005), the value of *L(K)* was not a suitable criterion for determining the optimal K, given that *L(K)* increased after reaching the real value of K in our data (see Fig. 5a). The coefficient of similarity between runs carried out with STRUCTURE for *K* = 4 was 0.988 ± 0.0042, indicating similar population genetic structure across the different replications (see Fig. 6). In Figure 7, the four groups inferred in the Bayesian analysis are also discriminated in the 3D PCA and the dendrogram, calculated with the Jaccard's similarity coefficient and clustered by UPGMA. It can be observed for our data that the genetic structure is consistent and reliable across different inference methods commonly employed in studies of differentiation and genetic diversity. Additionally, the consistency of the clustering estimated with the SESIM code was appropriate for the number of individuals and markers used in this study (173 × 244; SESIM-value = 0.0194) and similarly to what was reported by Medina et al. (2006), the number of markers had a great impact on the SESIM-value in our data.

It is important to consider that some areas of difficult access that have not been sampled may contain individuals of *L. origanoides*, for instance, the non-sampled area between the municipalities of Cepitá and Capitanejo (see Fig. 2). The same situation occurs between the municipalities of Socotá and Soatá in the medium and upper basins, respectively. Future studies should try to sample individuals from these areas and establish their relationship with the populations proposed here.

### Genetic diversity

Table 1 shows the different measures employed to quantitate the genetic diversity of *L. origanoides* in the canyon of the Chicamocha River. In general, levels of genetic diversity within populations were relatively high. The populations LBa, LBb, and MB exhibited similar levels of diversity across all the estimators used, varying between 0.285 and 0.319. The population UB showed the lowest genetic diversity (0.232–0.268) with a reduction of 20% compared with the other populations of the canyon. *Lippia origanoides* is considered to be a habitat specialist species, with high levels of plasticity and stress tolerance (Parra and Rodríguez 2007; Antolinez-Delgado and Rodríguez 2008; Camargo and Rodríguez 2008); however, we believe that probably the suboptimal conditions for the development of the species in the upper basin of the canyon are reflected in the lower levels of genetic diversity in this area. This scenario is consistent with the strong decline observed in the upper basin during the sampling phase with respect to the distribution and abundance of the species in the lower and medium basins, probably due to a reduction in population size by ecological factors (Byars et al. 2009).

**Table 1:** Estimates of genetic diversity calculated using different methods

			*H* (Nei)	*H* (Lynch and Milligan)				*H* (Panmictic heterozygosity)				*H* (Shannon)		Full loci		
							
Pop.	*N*	*P*	*H^‡^*	*H^§^*	SD	*H^¶^*	SD	*hs*	*Hs*	*Ht*	SD	*I*	SD	*P*	*H^‡^*	*I*
LBa	44	93.03	0.28702	0.29547	0.01096	0.30806	0.01030	0.29111	**-**	**-**	0.00569	0.4419	0.21130	64.79	0.19770	0.3073
LBb	29	87.70	0.28888	0.29368	0.01222	0.30838	0.01145	0.28819	**-**	**-**	0.00446	0.4251	0.24410	60.85	0.19890	0.2949
MB	66	93.03	0.28542	0.31120	0.01098	0.31953	0.01050	0.29176	**-**	**-**	0.00732	0.4444	0.22370	64.79	0.19640	0.3086
UB	34	75.41	0.23297	0.24934	0.01279	0.26807	0.01186	0.25096	**-**	**-**	0.00810	0.3486	0.26730	52.11	0.16030	0.2409
Mean	43.25	87.30	0.27357	0.28742	0.01174	0.30101	0.01103	**-**	0.28051	**-**	0.00553	0.4150	0.23660	60.63	0.18830	0.2879
Total	173	100	0.31999	0.33710	**-**	0.34370	**-**	**-**	**-**	0.31995	0.00209	0.4785	0.18920	71.27	0.22020	0.3330

*N*, Individuals by population; *P*, Percentage of polymorphic loci; *H^‡^*, Nei's gene divesity – *AFLPdat; H^§^*, Nei's gene divesity using Square root method *– AFLPsurv; H^¶^*, Nei's gene divesity using a Bayesian method with non-uniform prior distrbution *– AFLPsurv; hs*, Average panmictic heterozygosity within population – *Hickory; Hs*, Average of hs across populations – *Hickory; Ht*, Panmictic heterozygosity based on mean allele frequencies *– Hickory; I*, Shannon's information index – *PopGene*.

We also used the Shannon's information index (*I*) as an alternative measure to quantitate diversity (see Table 1). The populations LBa, LBb, and MB showed similar levels of diversity (*I* = 0.44) and significantly higher than UB (*I* = 0.348), which is in accordance to the results presented above. Interestingly, the genetic diversity in *L. origanoides* seems to follow a pattern previously reported for species inhabiting elevation gradients, which are characterized by exhibiting a marked decrease in genetic variation in marginal populations compared with populations centrally situated (Byars et al. 2009). Taking this pattern in mind, the population UB behaves as a marginal population in which different ecological factors have changed significantly reducing its population size and genetic diversity. In addition, in view of the fact that gene flow between populations or individuals in similar elevations is more likely than gene flow among those at different elevations (Byars et al. 2009), restricted gene flow may have been crucial in determining the current levels of genetic diversity in UB.

The individual contribution of each population to genetic diversity was determined using *Ht* (the panmictic heterozygosity in the total sample, based on mean allele frequencies), as suggested by Holsinger (1999). The contribution of each population (*k*) was estimated as *Ct* = (*Ht*−*Ht*/*k*)/*Ht* (Holsinger 1999), where *Ht*/*k* is the heterozygosity calculated when removing the population *k*. The results show a high contribution of population LBa to genetic diversity (1. *Ct*/LBa = 3.27%; 2. *Ct*/LBb = 0.18%; 3. *Ct*/MB = 0.53%; 4. *Ct*/UB = −0.54%).

The effect of monomorphic markers on estimation of diversity was evaluated. In Table 1, the columns headed *Full loci* show the values of Nei's genetic diversity and Shannon's information index estimated using the full set of markers scored in this study, 355 AFLP loci. The estimators of the diversity were reduced in approximately 30% compared with the data sets that fulfill the parameters of polymorphism, usually 0.99 level of polymorphism.

On the other hand, the genetic diversity of the population LBb (in the municipalities of Pescadero and Cepitá) was previously reported by Suárez et al. (2008) using ISSRs markers. In that study, the values of genetic diversity were *I* = 0.453 and *hs* = 0.484. Although the value of *I* reported for LBb in our study (*I* = 0.425) was similar to that obtained by Suárez et al. (2008), the value of *hs* presented here is significantly different (*hs* = 0.484 vs. *hs* = 0.288), probably due to the use of different molecular marker systems (Mariette et al. 2002).

### Population differentiation

The distribution of genetic diversity within and among populations in the canyon of the Chicamocha River was determined using two-level analysis of molecular variance (see Table 2). AMOVA showed highly significant genetic differences between populations (*F*_*ST*_ = 0.17916, *P-value* < 0.0000). In general, 17.9% of the variation was due to differences between populations of the canyon, while the remaining variation was due to differences between individuals within populations. The Bayesian estimate of differentiation among contemporary populations θ^II^ and implemented in HICKORY showed a similar level of population subdivision (θ^II^ = 0.15592 ± 0.02311). Pairwise *F*_*ST*_ estimates (see Table 3) indicated relatively high differentiation among distant populations (LBa−UB, *F*_*ST*_ = 0.306), and moderate differentiation between nearby populations (LBa−LBb, *F*_*ST*_ = 0.104; LBb−MB, *F*_*ST*_ = 0.132; MB−UB, *F*_*ST*_ = 0.123).

**Table 2:** Two-level analysis of molecular variance based on distance method of pairwise difference

Source of variation	df	Sum of squares	Variance components	Fixation indices	*P*-value	Percentage of variation
Among populations	3	1020.891	7.35344	0.17916	0.00000	17.92
Within populations	169	5693.837	33.69134			82.08
Total	172	6714.728	41.04478			

**Table 3:** Matrix of pairwise differentiation (*F*_*ST*_: lower diagonal; *P-value*: upper diagonal) based on distance method of pairwise difference

	LBa	LBb	MB	UB
LBa	-	0.00000	0.00000	0.00000
LBb	0.10403	-	0.00000	0.00000
MB	0.18700	0.13285	-	0.00000
UB	0.30651	0.22942	0.12302	-

Interestingly, the differentiation among populations decreased in 18% when the population LBa was removed and in 37% when the same procedure was performed for the population UB. This result is interesting because LBa and UB are located at the extremes of the distribution of *L. origanoides* in the canyon and at contrasting elevations (see Fig. 2), and there is also a high genetic differentiation between them. Thereby, these populations (LBa and UB) may be appropriate targets for research to understand the genetic basis of high tolerance to stress in xerophytic environments and local adaptation along an altitudinal gradient.

## Conclusions

In summary, with the results obtained in this study, we conclude that the genetic structure of *L. origanoides* in the canyon of the Chicamocha River is related to the geography of the area (lower, medium, and upper basins), and probably to the difference in altitude among the basins. The population that contributed the most to the genetic diversity was LBa, which corresponds to the municipalities of Juntas and Jordan, where further research and conservation activities should be focused. However, these previous results encourage further sampling and research in areas that have not been sampled yet in order to understand more of the adaptation and distribution of this important species in Colombia.
